# Proteome Analysis Related to Unsaturated Fatty Acid Synthesis by Interfering with Bovine Adipocyte *ACSL1* Gene

**DOI:** 10.3390/antiox13060641

**Published:** 2024-05-24

**Authors:** Yanbin Bai, Jingsheng Li, Yali Wei, Zongchang Chen, Zhanxin Liu, Dashan Guo, Xue Jia, Yanmei Niu, Bingang Shi, Xiaolan Zhang, Zhidong Zhao, Jiang Hu, Xiangmin Han, Jiqing Wang, Xiu Liu, Shaobin Li

**Affiliations:** Gansu Key Laboratory of Herbivorous Animal Biotechnology, College of Animal Science and Technology, Gansu Agricultural University, Lanzhou 730070, China; baiyb@st.gsau.edu.cn (Y.B.); lijs@st.gsau.edu.cn (J.L.); weiyl@st.gsau.edu.cn (Y.W.); chenzongc@st.gsau.edu.cn (Z.C.); liuzx@st.gsau.edu.cn (Z.L.); guods@st.gsau.edu.cn (D.G.); jiax@st.gsau.edu.cn (X.J.); niuym@st.gsau.edu.cn (Y.N.); shibg@gsau.edu.cn (B.S.); zhangxl@gsau.edu.cn (X.Z.); 893362306@163.com (X.H.); wangjq@gsau.edu.cn (J.W.); liux@gsau.edu.cn (X.L.); lisb@gsau.edu.cn (S.L.)

**Keywords:** ACSL1, unsaturated fatty acids, bovine adipocytes, proteome, long non-coding RNA

## Abstract

Unsaturated fatty acids (UFAs) in beef play a vital role in promoting human health. Long-chain fatty acyl-CoA synthase 1 (ACSL1) is a crucial gene for UFA synthesis in bovine adipocytes. To investigate the protein expression profile during UFA synthesis, we performed a proteomic analysis of bovine adipocytes by RNA interference and non-interference with *ACSL1* using label-free techniques. A total of 3558 proteins were identified in both the NC and si-treated groups, of which 1428 were differentially expressed proteins (DEPs; fold change ≥ 1.2 or ≤ 0.83 and *p*-value < 0.05). The enrichment analysis of the DEPs revealed signaling pathways related to UFA synthesis or metabolism, including cAMP, oxytocin, fatty acid degradation, glycerol metabolism, insulin, and the regulation of lipolysis in adipocytes (*p*-value < 0.05). Furthermore, based on the enrichment analysis of the DEPs, we screened 50 DEPs that potentially influence the synthesis of UFAs and constructed an interaction network. Moreover, by integrating our previously published transcriptome data, this study established a regulatory network involving differentially expressed long non-coding RNAs (DELs), highlighting 21 DEPs and 13 DELs as key genes involved in UFA synthesis. These findings present potential candidate genes for further investigation into the molecular mechanisms underlying UFA synthesis in bovines, thereby offering insights to enhance the quality of beef and contribute to consumer health in future studies.

## 1. Introduction

The fat deposition and unsaturated fatty acid (UFA) content in beef muscle not only impact the meat quality and flavor, but also contribute to enhancing the nutritional value of the meat. Studies have demonstrated that the UFAs present in beef, such as long-chain n-3 polyunsaturated fatty acids (PUFAs), docosahexaenoic acid (DHA; 22:6, n-3), and eicosapentaenoic acid (EPA; 20:5), play a crucial role in preventing cardiovascular disease and atherosclerosis, as well as promoting fetal brain and visual growth [1,2,3,4]. In addition, UFAs act as regulators of antioxidant activity and inflammatory responses [5,6]. As a member of the ACSL family (ACSL1, 3, 4, 5, and 6), long-chain acyl-CoA synthase 1 (ACSL1) is essential for fatty acid (FA) activation, transport, catabolism, and lipid synthesis [7,8]. Located on the outer mitochondrial membrane [7], *ACSL1* converts long-chain FAs into fatty acyl-CoA to facilitate triglyceride synthesis [9], stimulate FA deposition, and activate FAs [8] before finally entering the β-oxidation pathway [10]. A quantitative trait locus (QTL) study revealed that *ACSL1* exerts an influence on the relative levels of UFAs, omega-3 FAs, polyunsaturated FAs, and DHA [11]. Furthermore, *ACSL1* overexpression activates FA metabolism and facilitates their transportation for diglycerides and phospholipids instead of cholesterol esters [12]. In adipocytes, the arachidonic acid levels were further stimulated by the overexpression of *ACSL1* [13]. Moreover, our previous study demonstrated that *ACSL1* regulates UFA synthesis and lipid droplet production in bovine adipocytes, further suggesting that *ACSL1* is a key gene regulating UFAs [14,15,16].

Proteomic analysis is a robust technique for the investigation of protein expression patterns and has been extensively employed in studies involving sheep [17,18], chickens [19,20,21], and pigs [22,23]. It is worth noting that research on bovine FA metabolism from the perspective of transcriptomics or proteomics is still in its nascent stage. Currently, studies on bovine fat deposition or FA metabolism primarily focus on individual genes or related signaling axes, with a relatively limited emphasis on proteomics [24,25,26]. Poleti et al. [27] used label-free quantification to identify 1582 proteins in Nellore cattle’s longissimus dorsi muscle with high and low intramuscular fat (IMF) content. Studies have shown that proteins involved in changes in sarcomeric protein/mRNA levels, intracellular signaling, and the regulation of the actin cytoskeleton are involved in IMF deposition [27]. In a separate study, the longissimus dorsi muscle of Xinjiang brown cattle and Kazakh cattle underwent transcriptome and proteome sequencing, resulting in 22,677 transcripts and 1874 proteins [28]. Through joint analysis, 1737 genes were identified at both the transcriptome and proteome levels [28]. Additionally, Shen et al. [29] performed proteomic analysis by comparing Korean cattle with high and low skeletal muscle marbling scores, and the results preliminarily screened out some proteins that may regulate bovine IMF synthesis.

**Figure 1:**
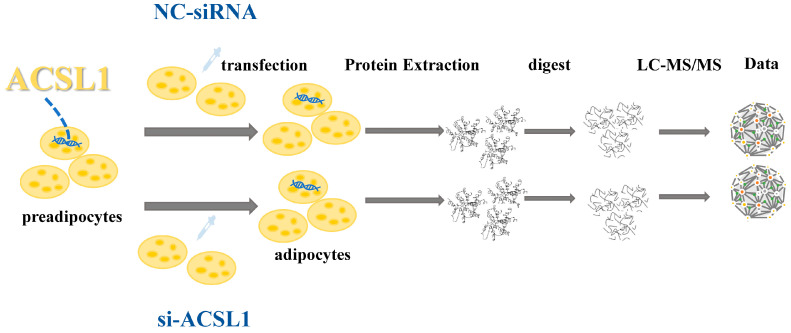
Label-free proteomic analysis test procedure.

In previous studies, we characterized the transcriptional profiles of long non-coding RNAs (lncRNAs) as well as mRNAs in bovine adipocytes after interference with the *ACSL1* gene, and we provided a network of functional interactions between the lncRNAs and protein-coding genes involved in UFA synthesis [30]. However, it is noteworthy that the protein levels and mRNA expression do not always exhibit a direct correlation [31,32], with proteins also providing valuable insights into post-translational modifications [33,34]. Additionally, lncRNAs regulate gene expression at the transcriptional and post-transcriptional levels [35,36], but they also regulate the transcription of downstream molecules by binding to proteins and localizing protein complexes to specific DNA sequences [37,38]. In this study, label-free proteomics analysis was performed using RNA interference and non-interference with *ACSL1* in bovine adipocytes (Figure 1). We screened the proteins in terms of UFA synthesis to understand their roles and modes in the regulation of UFA synthesis in bovine adipocytes. Furthermore, through a joint analysis with previous transcriptomic data, we aimed to identify the crucial lncRNAs and proteins that may be involved in UFA synthesis, as well as exploring the potential connections among them. These results provide a basis for the exploration of the genetic and molecular mechanisms of UFA synthesis in bovine adipocytes.

## 2. Materials and Methods

### 2.1. Ethics Statements

Perirenal adipose tissue was collected from a healthy 1-day-old bull calf born and raised at the animal farm of Gansu Agricultural University (Lanzhou, China) and subsequently slaughtered in a slaughterhouse. The study protocol was approved by the Gansu Agricultural University, China, as well as the Ministry of Science and Technology of the People’s Republic of China (approval number 2006–398). The animal experiments were approved by the Ethics Committee of Gansu Agricultural University (GSAU-2th-AST-2021-25) and complied with the local animal welfare regulations.

### 2.2. Sample Collection and Cell Culture

We collected perirenal adipose tissue from a 1-day-old bull calf at the Animal Farm of Gansu Agricultural University. The bovine precursor adipocytes were isolated and cultured according to the procedure described in our previous publication [15,30]. Briefly, the bovine perirenal fat was digested, filtered, and centrifuged before being resuspended and inoculated into 10 cm cell culture dishes. Subsequently, the cells were cultured at 5% CO_2_ and 37 °C until they reached 90% confluency, at which point they were processed.

### 2.3. Temporal Expression and siRNA Screening of ACSL1

*ACSL1* expression was assessed via qRT-PCR at various stages of bovine precursor adipocyte differentiation, specifically on days 0, 2, 4, 6, and 8. The siRNAs listed in Table 1 were designed and synthesized by the Guangzhou Ruibo Biological Company to provide their sequence information. The bovine adipocytes were transfected with si-ACSL1 and si-NC, respectively. The most effective siRNAs were identified by detecting *ACSL1* mRNA expression using qRT-PCR after 48 h. Finally, proteomic analysis was performed on bovine adipocytes transfected with the most efficient si-ACSL1 and si-NC.

### 2.4. Total Protein Extraction

The bovine adipocytes prepared at −80 °C were transferred to 1.5 mL centrifuge tubes. The samples for testing were labeled as si_ACSL1_1, si_ACSL1_2, and si_ACSL1_3 (*n* = 3) and NC_ACSL1_1, NC_ACSL1_2, and NC_ACSL1_3 (*n* = 3), respectively. An appropriate amount of DB protein solution containing 8 M urea and 100 mM TEAB with pH = 8.5 was added and mixed by shaking. After ultrasonic treatment in an ice water bath for 5 min to completely disrupt the cells, the cells were centrifuged at 4 °C at 12,000× *g* for 15 min, and the supernatant was collected. Subsequently, the supernatant was supplemented with 10 mM DTT and incubated at 56 °C for 1 h, followed by sufficient IAM treatment at 25 °C for 1 h.

### 2.5. Protein Inspection

The Bradford Protein Quantification Kit (Beyotime, Shanghai, China) was employed to prepare the BSA standard protein solution (BSA concentration gradient range 0–0.5 µg/L) according to the instructions. Various BSA standard protein solutions with distinct concentration gradients, as well as sample solutions with different dilution ratios, were dispensed into a 96-well plate up to a final volume of 20 µL (each gradient was repeated 3 times). Then, 180 µL of G250 staining solution was added and incubated at 25 °C for 5 min, followed by measuring the absorbance at 595 nm. The absorbance of the protein standard solution was used to construct the standard curve and calculate the protein concentration of the sample to be tested. Furthermore, 20 µg protein samples were subjected to electrophoresis on a 12% SDS-PAGE gel (the electrophoresis conditions of the separated gel were 120 V and 90 min, and the electrophoresis conditions of the concentrated gel were 20 min and 80 V). After the completion of electrophoresis, the R-25 Coomath bright blue stain was applied and destained until the bands were clear.

### 2.6. Enzymatic Hydrolysis of Protein

The protein sample was mixed with a protein solution containing 8 M urea and 100 mM TEAB at pH 8.5 to reach a final volume of 100 µL. Trypsin and 100 mM TEAB buffer were added and the mixture was digested at 37 °C for 4 h. This was followed by trypsin and CaCl_2_ enzyme digestion overnight. The mixture was mixed with formic acid until the pH was below 3. It was then centrifuged at 12,000× *g* at 25 °C for 5 min, and the supernatant was collected. The supernatant was then slowly passed through the C18 column for desalting. The cleaning solution (0.1% formic acid, 3% acetonitrile) was used three times consecutively. After this, an appropriate amount of eluent containing 0.1% formic acid and 70% acetonitrile was added to collect the filtrate, which was then freeze-dried.

### 2.7. Liquid Mass Detection

Mobile phase A consisted of 100% water and 0.1% formic acid, while mobile phase B consisted of 80% acetonitrile and 0.1% formic acid. The lyophilized powder (10 µL) from liquid A was centrifuged at 14,000× *g* at 4 °C for 20 min, followed by the extraction of a supernatant sample (1 µg) for liquid quality analysis. The UHPLC system was upgraded with easy-NLCTM 1200 nA (Thermo Fisher scientific, Waltham, MA, USA). The precolumn was self-made and measured 4.5 cm × 75 μm with 3 μm particles. The analytical column was also self-made and measured 15 cm × 150 μm with 1.9 μm particles. The elution conditions for liquid chromatography are shown in Table 2. A QexactiveTM HF-X mass spectrometer (MS) with a Nanospray Flex™ (ESI) ion source (Thermo Fisher scientific, Waltham, MA, USA) was used. The ion spray voltage was set to 2.1 kV and the ion transfer tube temperature was maintained at 320 °C. The MS acquisition mode employed in this study was data-dependent, with a comprehensive scanning range of *M*/*Z* 350–1500 and a first-level resolution of 60,000 (200 *m*/*z*). The maximum injection time for the C-trap was set at 20 ms, while its maximum capacity was 3 × 10^6^. Subsequently, the top 40 parent ions identified during the full scan were selected for fragmentation using high-energy collision dissociation (HCD) and subsequently detected by secondary MS analysis. For the secondary MS analysis, a resolution of 15,000 (200 *m*/*z*) was utilized along with a maximum C-trap injection time of 45 ms and a maximum C-trap capacity of 1 × 10^5^ ions. The peptide fragmentation collision energy was maintained at 27%, while the threshold intensity was set to 2.2 × 10^4^. Additionally, dynamic exclusion with a range of 20 s was implemented to avoid repeated detection within this timeframe. Finally, the raw MS detection data were generated.

### 2.8. Identification and Quantification of Proteins

The protein database Bos taurus (Bovine) from Uniprot (containing 46,725 sequences) was used to search all result spectra through Proteome Discoverer 2.2 (PD2.2, Thermo). The search parameters were established via following steps: (1) the precursor ion mass tolerance was set to 10 parts per million (PPM); (2) the fragment ion mass tolerance was set to 0.02 Daltons (Da); (3) cysteine alkylation was employed as the immobilized modification; (4) methionine oxidation was utilized as the modification method; (5) acetylation was applied for N-terminus modification; (6) up to 2 missed cleavage sites were permitted.

To enhance the quality of the analysis results, we employed the Proteome Discoverer software (Version 2.2) for the further refinement of the search outcomes. (1) Peptide spectrum matches (PSMs) with reliability exceeding 99% were considered credible; (2) proteins containing at least one unique peptide segment were also regarded as credible; (3) FDR verification was conducted on credible spectrum peptides and proteins, and any with an FDR > 1% were eliminated; (4) T-test analysis was utilized to identify significantly different proteins between the NC and si-treated groups. Proteins with a *p*-value ≤ 0.05 and fold change (FC) ≥ 1.2 or FC ≤ 0.83 were defined as differentially expressed proteins (DEPs). Upregulated DEPs were defined as those with FC ≥ 1.2 and a *p*-value ≤ 0.05, while downregulated DEPs were defined as those with FC ≤ 0.83 and a *p*-value ≤ 0.05.

### 2.9. Cluster and Functional Enrichment Analysis of DEPs

A cluster analysis was used to analyze the relative content of DEPs in each sample. A cluster heatmap was used to observe the upregulation and downregulation of DEPs when comparing different samples. The clustering heatmap was generated using the R language and the R package pheatmap, version 3.4.3, based on protein quantification values (https://cran.r-project.org/web/packages/pheatmap/index.html/ (accessed on 22 July 2023)). Each row was Z-corrected, meaning that the observed value was subtracted by the row mean and then divided by the row standard deviation. The Interproscan software (Version 5.0) was utilized for Gene Ontology (GO) functional annotation (http://geneontology.org/ (accessed on 26 July 2023)), while the Kyoto Encyclopedia of Genes and Genomes (KEGG) was employed for the functional protein family and pathway analysis of the identified proteins (http://www.genome.jp/kegg/ (accessed on 26 July 2023)) [39,40]. The KEGG pathway functional annotations included biological processes (BP), cellular components (CC), and molecular functions (MF). A *p*-value < 0.05 indicated a significant relationship between the terms and DEPs.

### 2.10. Construction of DEP Interaction Network

The interaction of the DEPs that were identified through the KEGG enrichment analysis in UFA synthesis or metabolism-related pathways was analyzed using the STRING DB software (Version 12.0) [41]. An interaction network was constructed using the software, with Bos taurus (cattle) selected as the species and an interaction score threshold of > 0.4 (moderate confidence) based on the requirements (http://STRING.embl.de/ (accessed on 30 July 2023)) [42]. Each node in the interaction network represents a protein, while the edges represent the strength of the interactions between proteins. The number of edges reflects the level of confidence in these interactions.

### 2.11. Combination of Transcriptome Data to Construct DEL-DEP Regulatory Network

LncRNAs bind to proteins and regulate the transcription of downstream molecules by localizing protein complexes to specific DNA sequences [37,43]. This transcriptional regulation influences gene expression through both cis-acting and trans-acting mechanisms [37,43]. Our study integrated previously published lncRNA and mRNA sequencing data and conducted a comprehensive transcriptome–proteome analysis using the Cytoscape (Version 3.6.0) software. Specifically, the DEL-DEP regulatory network, which involved lncRNA cis-targets, was constructed by intersecting the cis-acting targets of the DELs in the transcriptome results [30] with the DEPs in the proteomic data. Similarly, the trans-targets of the DELs in the RNA-seq results [30] were intersected with the DEPs in the proteomic data. To enhance the clarity, these findings were intersected with DEMs, resulting in the construction of the DEL-DEP regulatory network.

## 3. Results

### 3.1. ACSL1 Temporal Expression and Effective siRNA Screening

The expression trend of *ACSL1* exhibited an initial increase followed by a subsequent decrease on days 0, 2, 4, 6, and 8 of bovine preadipocyte differentiation. Notably, the highest expression level was observed on day 4 (*p* < 0.01) [15,30]. The transfection of three pairs of siRNAs on day 4 of bovine adipocyte differentiation showed that si3-ACSL1 had the highest interference efficiency (86%; *p* < 0.01) [15,30]. Consequently, we performed proteomic analysis by transfecting adipocytes with si3-ACSL1 (si-treated group) and si-NC on day 4 of differentiation.

### 3.2. Determination of Protein Extracted from Bovine Adipocytes

**Figure 2:**
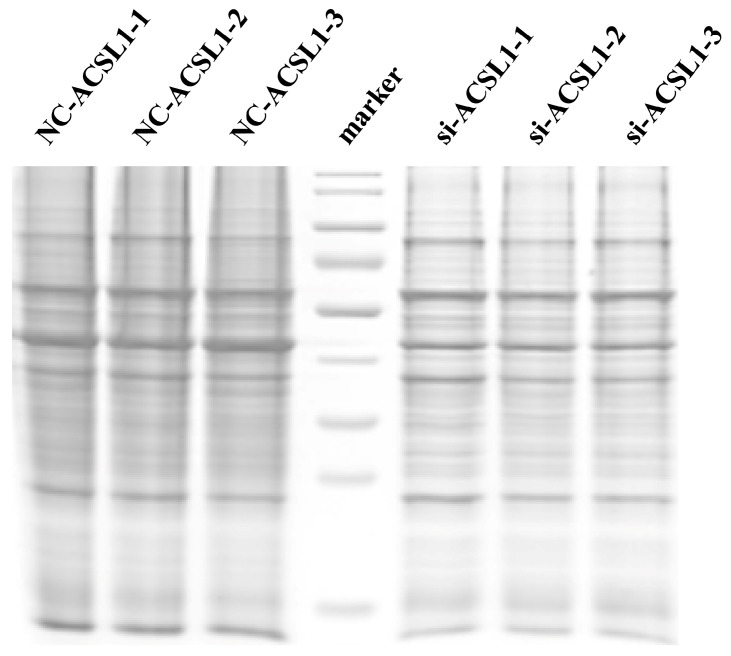
SDS-PAGE electrophoresis detection results of proteins extracted from bovine adipocytes (si-ACSL1, NC-ACSL1).

Statistical information on the proteins in the quantitative samples is shown in Table 3. The average total protein content of the bovine adipose cells in the NC-treated group (NC-ACSL1-1, NC-ACSL1-2, and NC-ACSL1-3) and si-treated group (si-ACSL1-1, si-ACSL1-2, and si-ACSL1-3) exceeded 100 μg and 50 μg, respectively. The protein concentration and total amount met the requirements of the subsequent assays. Moreover, 3 μL protein was extracted from each treatment and subjected to SDS-PAGE analysis. The resulting electrophoretic bands exhibited clear, abundant, and homogeneous patterns suitable for further experimental investigations (Figure 2).

### 3.3. Overview of Proteomics Information

To identify and screen proteins associated with the regulation of UFA synthesis in bovine adipocytes, this study employed the same samples as previously published regarding transcriptome sequencing (i.e., si-ACSL1- and NC-ACSL1-treated bovine adipocyte samples, each with three replicates) for label-free proteomic analysis [30]. The retrieval results were further filtered using peptide spectrum matches (PSMs) with reliability greater than 99% as trusted PSMs and proteins containing at least one unique peptide were considered trustworthy to enhance the quality of the analysis results and reduce the false positive rate. Only peptides and proteins that were deemed trustworthy were retained, and FDR validation was conducted to eliminate peptides and proteins with FDR > 1%. The data analysis yielded 400,282 secondary spectra, of which 140,710 were considered valid. When the confidence level exceeded 99%, a total of 35,802 peptides were identified and they contained at least one specific peptide. Furthermore, 3558 proteins were identified with at least one unique peptide when the confidence levels were above 99% and the FDR was less than 1% (Table 4).

Additionally, the distribution of peptides with varying lengths was analyzed. The peptide length ranged from 1 to 52 amino acid residues, with a predominant range of 7 to 24 amino acid residues. The number of peptides containing 12 amino acid residues was the largest, followed by those containing 11 and 10 amino acid residues. Peptides containing less than 40 amino acid residues were few (Figure S1). In addition, the number of proteins containing one unique peptide was the largest in this study, with a total of 521 proteins, and there were 501 proteins that contained two unique peptides. Generally, the number of proteins decreased as the number of matched unique peptides increased, and the number of proteins containing more than 67 unique peptides increased by single digits (Figure S2). Moreover, all identified proteins in this study were categorized into 11 classes based on their protein molecular weight at 10 kDa intervals (total protein number of proteins was 140,710; number of identified proteins was 3558) (Figure S3).

### 3.4. Screening of DEPs

**Figure 3:**
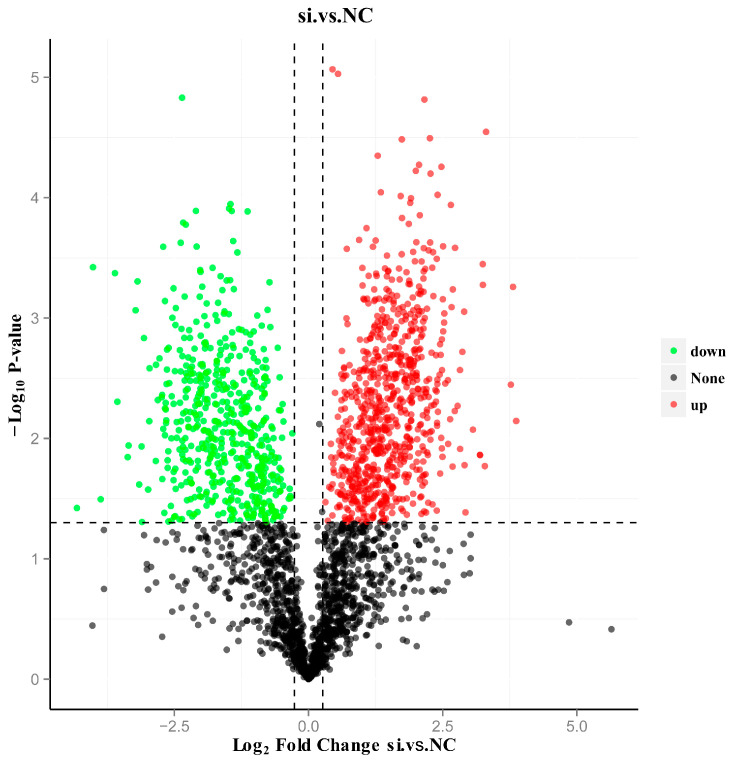
Volcano plot of DEP distribution in NC-treated group vs. si-treated group. The *x*-axis represents the difference fold (log2 value) of the differential proteins, the *y*-axis represents the *p*-value (−log10 value), black represents the proteins with no significant difference, red represents the upregulated proteins, and green represents the downregulated proteins.

In this study, 2762 proteins were identified in the samples from both the NC- and si-treated groups. Among them, 1428 DEPs were screened using a significance threshold of *p*-value ≤ 0.05 and FC ≥ 1.2 or ≤ 0.83 (Table S1). Specifically, we identified 809 upregulated proteins with FC ≥ 1.2 and a *p*-value ≤ 0.05, as well as 619 downregulated proteins with FC ≤ 0.83 and a *p*-value ≤ 0.05. The logarithm base 2 of the difference multiple of each protein was taken, and the absolute value of the *p*-value was taken to the logarithm base 10 to obtain the volcano plot of the DEPs (Figure 3).

### 3.5. Cluster Analysis of DEPs

**Figure 4:**
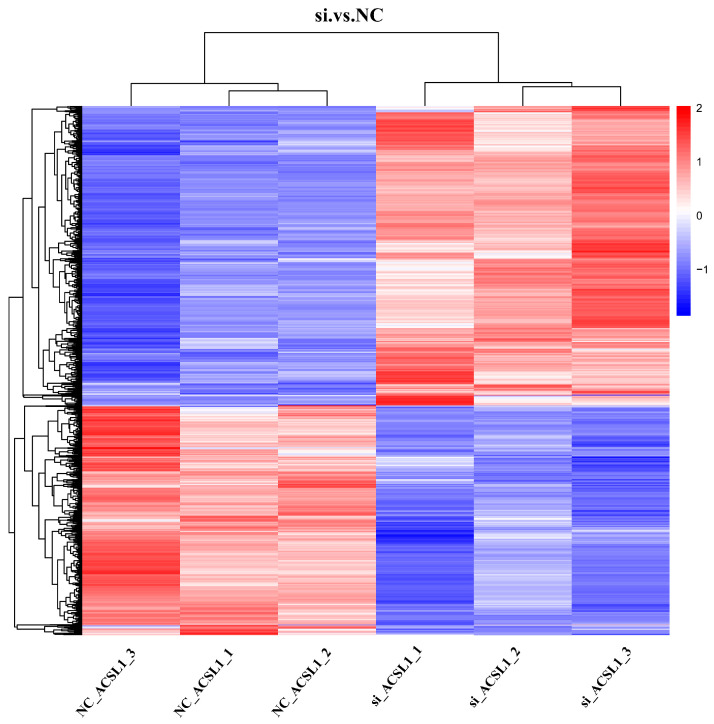
Heatmap of differential protein clustering of DEPs in NC-treated group vs. si-treated group. Red represents upregulation; blue represents downregulation; vertical is the clustering of samples; horizontal is the protein clustering; the shorter the clustering branches, the higher the similarity; the vertical clustering shows the expression pattern of the protein content between the sample clustering.

To further verify the reliability and reproducibility of this study, hierarchical clustering analysis was conducted on the DEPs identified from the proteomic data screening. The results are depicted in Figure 4, which shows the longitudinal clustering of the two experimental treatments of bovine adipocytes, and the transverse clustering of the DEPs is also shown. The si and NC treatments of the *ACSL1* gene in bovine adipocytes showed the same color in each of the three experimental replicates. The longitudinal clustering analysis revealed that the protein expression patterns exhibited similarity between the two experimental treatments, resulting in their grouping into a single cluster. Furthermore, both the horizontal and vertical clustering models demonstrated substantial inter-group differences in the proteomic data, while exhibiting excellent intra-group repeatability. These findings underscore the high accuracy and reliability of the samples analyzed in this study.

### 3.6. GO Enrichment Analysis of DEPs

**Figure 5:**
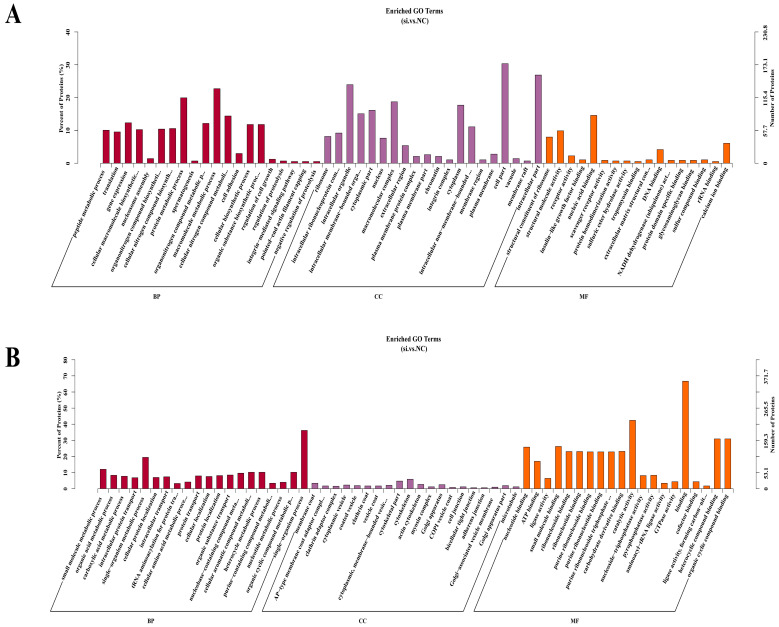
GO enrichment analysis of DEPs. (**A**) Top 50 GO entries significantly enriched by upregulated DEPs (including biological processes, cellular components, and molecular functions). (**B**) Top 50 GO entries significantly enriched by downregulated DEPs (including biological processes, cellular components, and molecular functions).

The GO enrichment analysis results showed that a total of 503 functional GO terms were significantly enriched by upregulated DEPs, with 74 terms showing significant enrichment (*p*-value < 0.05; Table S2). Figure 5A illustrates the top 50 functional GO terms that were significantly enriched by upregulated DEPs. In terms of biological processes (BP), the upregulated DEPs exhibited significant enrichment in 29 GO terms (*p*-value < 0.05), including protein metabolism (GO: 0019538), cell biosynthesis (GO: 0044249), cell growth regulation (GO: 0001558), lipid transport (GO: 0006869), and the negative regulation of cellular processes (GO: 0048523). Regarding cell component (CC)-related GO terms, the upregulated DEPs showed significant enrichment in 28 GO terms (*p*-value < 0.05), with the top three GO items being ribosomes (GO: 0005840), the intracellular ribonucleoprotein complex (GO: 0030529), and organelles (GO: 0043229). In molecular function (MF)-related GO terms, the upregulated DEPs demonstrated significant enrichment in 17 GO terms (*p*-value < 0.05), primarily associated with binding and enzyme activity.

A total of 570 functional GO terms were enriched by downregulated DEPs, of which 177 terms were significantly enriched (*p*-value < 0.05; Table S3). Figure 5B illustrates the top 50 functional GO terms significantly enriched by downregulated DEPs. In BP-related GO terms, the downregulated DEPs were significantly enriched in 75 GO terms (*p*-value < 0.05). These included organic acid metabolism (GO: 0006082), pyruvate metabolism (GO: 0006090), glycolysis (GO: 0006096), precursor metabolite and energy production (GO: 0006091), and gluconeogenesis (GO: 0006094). In CC-related GO terms, the downregulated DEPs showed significant enrichment in 21 GO terms (*p*-value < 0.05), primarily associated with the protein junction complex (GO:0030131; GO:0030119), cytoskeleton (GO: 0044430; GO: 0005856; GO: 0015630), and Golgi apparatus (GO: 0005794; GO: 0044431). In the MF-related GO terms, the DEPs were significantly enriched in 81 GO terms (*p*-value < 0.05), which were mainly related to binding, enzyme activity, and acceptors.

### 3.7. KEGG Enrichment Analysis of DEPs

**Figure 6:**
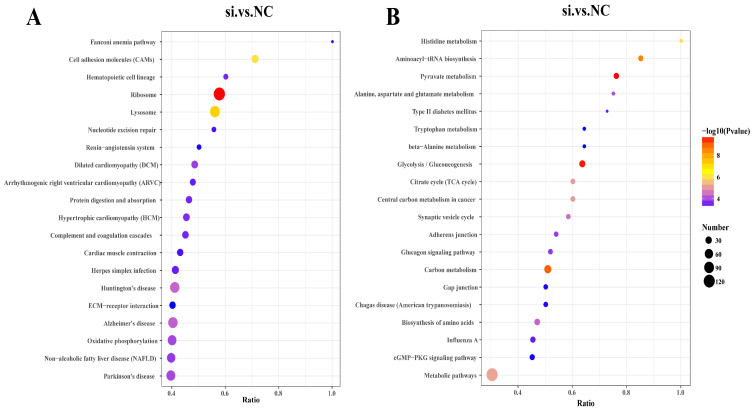
KEGG enrichment analysis of DEPs. (**A**) Analysis of the top 20 KEGG signaling pathways enriched by upregulated DEPs. (**B**) Analysis of the top 20 KEGG signaling pathways enriched by downregulated DEPs. The figure’s *x*-axis represents the ratio of differential proteins in the pathway to the total number of identified proteins. A higher value indicates the greater enrichment of differential proteins in the pathway. The point’s color represents the *p*-value of the hypergeometric test, ranging from blue to red. The color red indicates a smaller value, which means that the test is more reliable and statistically significant. The size of the dot corresponds to the number of differential proteins in the pathway, with larger dots representing more differential proteins.

The KEGG enrichment analysis revealed that the upregulated and downregulated DEPs were involved in 82 and 206 signaling pathways, respectively. Among these pathways, the upregulated DEPs were significantly enriched in 17 signaling pathways (*p*-value < 0.05; Table S4), including ribosomes, lysosomes, cell adhesion molecules, oxidative phosphorylation and protein digestion, and absorption signaling pathways (Figure 6A). Furthermore, we identified certain upregulated DEPs associated with UFA synthesis-related signaling pathways, such as arachidonic acid metabolism, ECM–receptor interaction, and the PI3K–Akt signaling pathway. Although these proteins are not significantly enriched in signaling pathways associated with upregulated DEPs, they may still contribute to UFA synthesis.

The downregulated DEPs were significantly enriched in 73 signaling pathways (*p*-value < 0.05; Table S5). Among the top 20 pathways were pyruvate metabolism, glycolysis/gluconeogenesis, the citric acid cycle (TCA cycle), aminoacyl-tRNA biosynthesis, and amino acid biosynthesis (Figure 6B). It is important to note that certain downregulated DEPs were significantly enriched in signaling pathways related to UFAs synthesis or metabolism, including the cAMP signaling pathway, the oxytocin signaling pathway, fatty acid degradation, glycerol metabolism, the insulin signaling pathway, and the regulation of lipolysis in fat cells (*p*-value < 0.05). Table 5 presents the UFA synthesis or metabolism signaling pathways that were significantly enriched by downregulated DEPs in this study.

### 3.8. Interaction Networks of DEPs in Signaling Pathways Related to Synthesis of UFAs

Proteins typically do not function in isolation but rather interact with one another to carry out diverse biological processes. In this study, we screened the DEPs exhibiting the significant enrichment of UFA synthesis or metabolism-related signaling pathways, including cAMP signaling pathways, oxytocin signaling pathways, fatty acid degradation, glyceride metabolism, fat cells in the regulation of lipolysis, and insulin signaling pathways. We focused on the significant enrichment of 50 DEPs and finally constructed a protein interaction network for UFA synthesis and metabolism.

**Figure 7:**
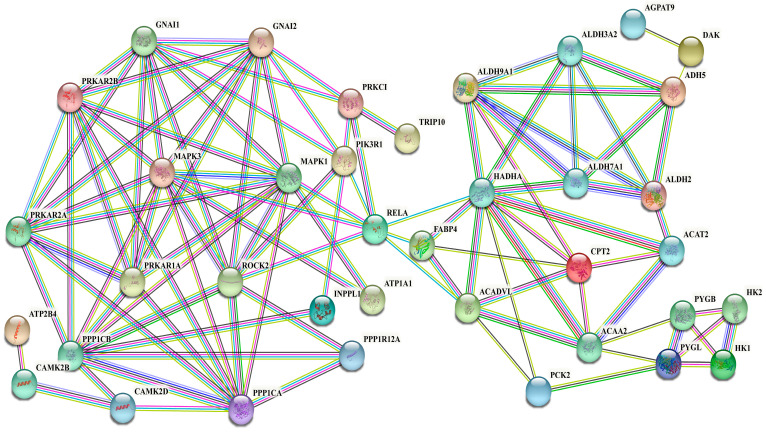
DEP interaction network of UFA synthesis-related signaling pathways. Each node in the interaction network represents a protein, and the edges represent the strength of the interaction between the proteins. The number of edges indicates the level of confidence in the interaction.

Most DEPs in the protein interaction network have strong interaction relationships (Figure 7), such as the fatty acid metabolism-related proteins ALDH9A1, ALDH7A1, ALDH3A2, ALDH2, HADHA, ACAA2, and ACAT2. Extremely long-chain specific acyl-CoA dehydrogenase (ACADVL) was strongly correlated with potential fatty acid synthesis-related proteins such as RELA, MAPK1, MAPK3, PIK3R1, and PABP4. Additionally, the phosphorylation-related proteins ROCK2, MAPK1, MAPK3, PPP1CA, PPP1CB, and PPP1R12A had strong interactions with the signal transduction guanosine binding proteins GNAI1 and GNAI2 and the protein kinases PRKAR2A and PRKAR2B. Moreover, certain proteins, including AGPAT9, DK2, TRIP10, and ATP2B4, were located at the periphery of the interaction network.

### 3.9. lncRNA–Protein Regulatory Network Construction Combined with Transcriptome Data

**Figure 8:**
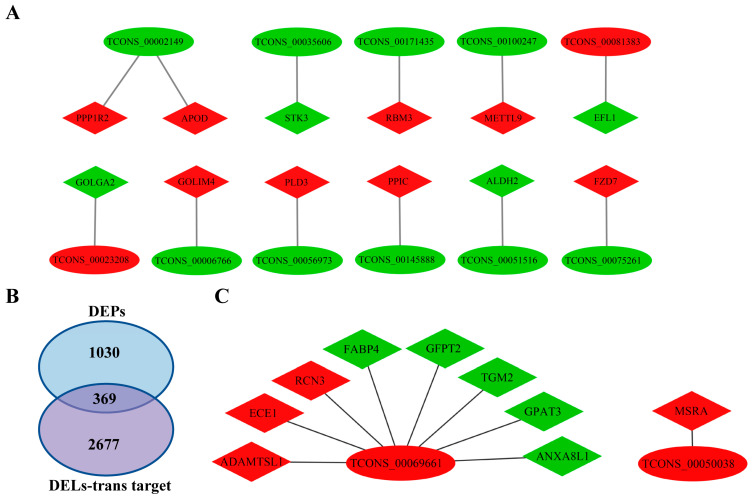
lncRNA–protein regulatory network. (**A**) DEL-DEP (lnRNA cis-target) interaction network. (**B**) Intersection of DEL trans-targets and proteomic DEPs. (**C**) DEL-DEP (lnRNA trans-target) interaction network. Ovals represent differentially expressed lncRNAs; diamonds represent differentially expressed proteins; red represents differential upregulation; green represents differential downregulation.

One of the modes of action of lncRNAs is that they can bind to proteins, leading to the targeting of protein complexes to specific DNA sequences and the subsequent regulation of downstream molecule transcription. The mode of transcriptional regulation can influence gene expression via both cis and trans mechanisms [37,43]. In this study, we integrated the previously published lncRNA RNA-seq data to construct the lncRNA–protein regulatory network between differentially expressed lncRNAs (DELs) and DEPs [30]. First, the cis-targets of DELs in the transcriptome data were intersected with the DEPs to construct the DEL-DEP (lnRNA cis-target) regulatory network (Figure 8A; Table S6). Within this interaction network, 11 DELs and 12 DEPs may have potential regulatory relationships, such as TCONS_00002149 and PPP1R2 and APOD, TCONS_00081383, and EFL1.

Additionally, by integrating the lncRNA and mRNA RNA-Seq data [30], the trans-targets of the DELs were first intersected with the DEPs, resulting in 369 common target genes (Figure 8B; Table S7). To enhance the clarity of the interaction network, these results were further intersected with differentially expressed mRNAs, leading to the construction of a DEL-DEP (lnRNA trans-target) regulatory network (Figure 8C). In this constructed DEL-DEP (lncRNA trans-target) interaction network, it was observed that two DELs and nine DEPs may have a potential regulatory relationship; for example, TCONS_00069661 interacts with ADAMTSL1, ECE1, RCN3, FABP4, GFPT2, TGM2, GPAT3, and ANXA8L1, and TCONS_00050038 interacts with MSRA. In conclusion, these DEPs and their interacting DELs may belong to the core proteins or lncRNAs that regulate UFA synthesis.

## 4. Discussion

The levels of UFAs in beef play a crucial role in both human health and the beef industry, impacting the beef quality, nutritional content, and carcass value. In recent years, numerous studies have investigated the mRNA expression in bovine muscle and adipose tissue to elucidate the molecular mechanisms underlying intramuscular fat (IMF) deposition and FA metabolism. These studies have identified hundreds of genes involved in various biochemical pathways and cell signaling mechanisms [44,45]. However, it is important to note that research on bovine fat deposition and FA metabolism has primarily concentrated on individual genes or associated pathways, with limited proteomic-based studies available [24,25,26]. Notably, the mRNA expression levels related to fat deposition or FA metabolism did not exhibit a strong correlation with the protein abundance [32,46]. In this study, we aimed to investigate the expression of the key gene *ACSL1*, which is involved in the production of UFAs in bovine adipocytes. Additionally, we systematically identified proteins that regulated UFA synthesis in bovine adipocytes after different treatments of *ACSL1* (NC-ACSL1, si-ACSL1) using label-free technology.

A total of 3240 proteins were identified from the NC- and si-treated groups, each with at least one unique peptide segment. Among these, 1428 proteins exhibited differential expression (*p*-value ≤ 0.05 and FC ≥ 1.2 or ≤ 0.83). The cluster analysis results of the differentially expressed proteins (DEPs) revealed that the same group of DEPs within the same group clustered together, demonstrating consistent expression patterns in line with the differential analysis and ensuring the high accuracy and reliability of the samples. Additionally, the peptide length distribution, number of unique peptides, and protein molecular weight distribution in the proteome analysis met the research conditions. Notably, this study detected a higher number of DEPs (1428) compared to previous studies [17,27,28,47], which can be attributed to variations in the screening thresholds employed for the differential analysis. The screening threshold used in some studies may be too high, resulting in the exclusion of important genes or proteins [20,27]. Conversely, setting the screening threshold too low for differential analysis may prolong the filtration process of irrelevant information during analysis. Nevertheless, this approach could potentially unveil a greater number of key genes or proteins [17,47]. Furthermore, the expression pattern of the DEPs observed in this study aligns with previous research findings, suggesting that differential proteins between different species have comparable expression patterns. For a single protein, the majority either upregulate or downregulate its expression level and then participate in fat/UFA synthesis.

The GO analysis divided the significantly enriched upregulated DEPs (*p*-value < 0.05) into 74 functional groups (including 29 BPs, 28 CCs, and 17 MFs) and the significantly enriched downregulated DEPs (*p*-value < 0.05) into 177 functional groups (including 29 BPs, 75 CCs, and 81 MFs). The KEGG enrichment analysis revealed that the upregulated DEPs were associated with a total of 82 pathways, while the downregulated DEPs were involved in 206 pathways in total. Among them, upregulated DEPs were significantly enriched in 17 pathways, and downregulated DEPs were significantly enriched in 73 signaling pathways (*p*-value < 0.05). Importantly, some DEPs were enriched in potential signaling pathways of UFA synthesis or metabolism, such as the PI3K–Akt signaling pathway, arachidonic acid metabolism, ECM–receptor interaction, the cAMP signaling pathway, the oxytocin signaling pathway, fatty acid degradation, glycerol lipid metabolism, the insulin signaling pathway, and the regulation of lipolysis in adipocytes. In these aforementioned pathways, we screened 72 DEPs that were significantly enriched, such as ALDH9A1, ALDH2, ACAA2, MAPK1, MAPK3, PABP4, and PIK3R1.

The activation of the insulin signaling pathway has been reported to occur through the binding of insulin to the tyrosine kinase receptor insulin receptor (IR) [48]. Most of the metabolic effects of IR are mediated by signaling involving the phosphorylation of insulin receptor substrate 1 (IRS1) and the FoxO1 gene, protein kinase B (Akt), and the phosphatidylinositol 3-kinase (PI3K) activation pathway [49,50]. In this study, the DEPs significantly enriched in the insulin signaling pathway included PYGL, HK1, PCK2, PPP1CB, PRKAR2A, PPP1CA, MAPK1, MAPK3, HK2, PYGB, PRKAR1A, PRKAR2B, TRIP10, PRKCI, INPPL1, and PIK3R1. The PI3K–Akt signaling pathway represents a prototypical insulin-related signaling pathway [51]. PI3K plays a crucial role in protein phosphorylation, while the PI3K–Akt pathway is also implicated in adipogenesis [52,53,54]. The insulin-mediated PI3K–Akt signaling pathway played a critical role in the adipocytes of obese mice (db/db mice), resulting in excess lipids that needed to be properly stored in the adipose tissue [52]. In addition, the activation of the Akt pathway is believed to modulate the expression of PPARγ and C/EBPα during adipogenic processes, thereby promoting/repressing adipocyte adipogenicity and differentiation [53,55,56]. In this study, the DEPs A0A3Q1MXS2, A0A452DIS2, F1MTN1 F1MK44, P62261, P63103, A0A140T894, A0A3Q1MQS3, Q5E995, Q5EAC6, F1MYS7, Q3ZBS7, P80747, Q7SIB2, Q0P594, A0A3Q1MPS8, Q3T0F9, A0A3Q1MT97, Q0P5A7, A0A3Q1LWY4, A1A4L1, F1MUT9, and A0A452DK9 were enriched in the PI3K–Akt signaling pathway, suggesting that these proteins may induce FA-related metabolic pathways to synthesize UFAs in cattle. The ECM is critical for the tissue architecture and plays an important role in adipogenesis [57]. The ECM is abundantly enriched in bovine adipose tissue and has the ability to regulate specific genes involved in adipogenesis [58]. In this study, 12 DEPs were significantly enriched in the ECM–receptor interaction signaling pathway, including A0A3Q1MXS2, A0A452DIS2, F1MTN1, F1MK44, Q3ZBS7, P80747, Q7SIB2, A0A3Q1MPS8, Q29423, A0A3Q1MT97, A0A3Q1MFX6, and A0A3Q1MNL3. In conclusion, these DEPs screened through GO and KEGG enrichment analysis may exert a crucial influence on UFA synthesis or lipid metabolism by modulating their phosphorylation levels in the transduction pathways associated with UFA synthesis.

The protein interaction network comprises interactions between proteins and participates in various life processes, including biological signal transmission, gene expression regulation, energy and material metabolism, and cell cycle regulation. The analysis of protein interactions in biological systems is crucial in understanding protein function, energy metabolism, and functional connections. In mammals, the synthesis of UFAs is intricate and involves crucial proteins [59]. This study presents a comprehensive protein interaction network elucidating UFAs’ synthesis and metabolism by utilizing 50 DEPs, which were significantly enriched in various pathways, including the cAMP signaling pathway, oxytocin signaling, fatty acid degradation, glycerol lipid metabolism, lipolysis in adipocytes, and the insulin signaling pathway. The protein interaction network includes several DEPs with strong interactions, such as ALDH9A1, ALDH7A1, ALDH3A2, ALDH2, ALDH3A2, HADHA, ACAA2, and ACAT2, which are related to fatty acid metabolism. The protein ACADVL, which is specific to very-long-chain acyl-CoA dehydrogenase, is strongly associated with RELA, MAPK1, MAPK3, PIK3R1, and PABP4. These proteins are potentially involved in fatty acid synthesis.

Aldehyde dehydrogenases (ALDHs) are a class of enzymes that catalyze the irreversible oxidation of aliphatic and aromatic aldehydes using NAD(P). The genomes of eukaryotic organisms harbor a total of 24 distinct family members belonging to the ALDH family [60]. In this study, we screened the following ALDHs in the cAMP signaling pathway: ALDH9A1, ALDH7A1, ALDH3A2, ALDH2, ALDH3A2, and ACAT2. ALDHs are known to play a pivotal role in FA metabolism [61]. We hypothesize that the disruption of the *ACSL1* gene inhibits the metabolism of UFAs, leading to a reduction in the gene expression level of ALDHs enriched in the cAMP signaling pathway and subsequently reducing the phosphorylation level of the cAMP signaling pathway in UFA metabolism. Fatty acid beta-oxidation (FAO) is the primary process by which fatty acids are oxidized and serves as a primary energy source for the heart and skeletal muscle [62,63]. FA molecules within the mitochondrial matrix are necessary to initiate the process of fatty acid β-oxidation. This process involves the entry of fatty acids into the β-oxidative helix, leading to the generation of multiple acetyl-CoA molecules. ATP is subsequently produced via the tricarboxylic acid (TCA) cycle and the electron transport chain [64]. The mitochondrial pathway for fatty acid β-oxidation comprises four enzymatic steps catalyzed by acyl-CoA dehydrogenase very long chain (ACADVL), enoyl-CoA hydratase/3-hydroxyacyl-CoA dehydrogenase (EHHADH), hydroxy acyl-CoA dehydrogenase (HADHA), and acetyl [65]. The study revealed a strong correlation between ACADVL and potential fatty acid synthesis-related proteins, including RELA, MAPK1, MAPK3, PIK3R1, PABP4, and HADHA. These DEPs may facilitate the uptake of fatty acids and subsequent β-oxidation through protein interactions during UFA synthesis in bovine adipocytes.

**Figure 9:**
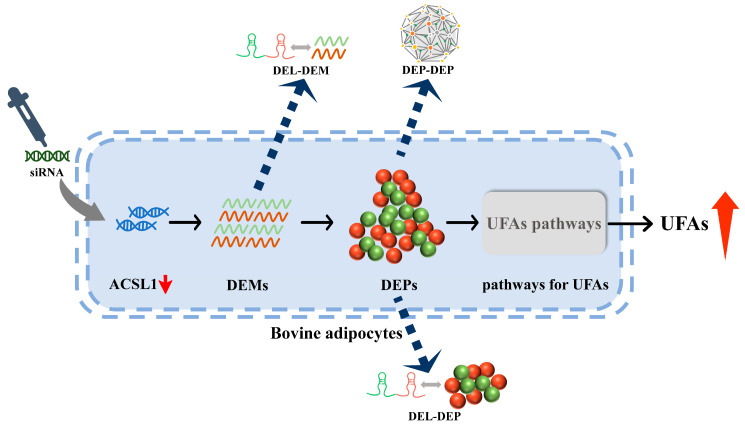
Mechanisms of DEP and DEL involved in UFA synthesis.

Furthermore, this study screened DEPs from the cAMP signaling pathway, such as PPP1R12A, PPP1CB, PPP1CA, MAPK1, and MAPK3, which also exhibit strong interactions. Although some of the significantly enriched proteins have not yet been characterized in adipocyte lipid metabolism and UFA synthesis, the cAMP signaling pathway itself plays a crucial role in these processes. Mature adipocytes are derived from fibroblast precursors through a dynamic differentiation process that necessitates extensive chromatin remodeling [66]. The cAMP signaling pathway is a well-characterized mechanism governing adipocyte differentiation [67,68]. An initial elevation in adipocyte cAMP levels stimulates PKA, which then phosphorylates and activates the nuclear basic leucine zipper transcription factor cAMP response element binding (CREB) protein and ATF family members [67,68]. These transcriptional activators are associated with pivotal regulators that induce adipogenesis, including PPAR-γ, C/EBPα, and C/EBPβ [68,69,70]. Therefore, we propose that the aforementioned DEPs may exert regulatory control over lipid metabolism and UFA synthesis by interacting with proteins enriched in the cAMP signaling pathway (Figure 9).

In recent years, mounting evidence has shown that non-coding RNAs, such as long non-coding RNAs (lncRNAs), microRNAs, and circular RNAs, play a significant role in various adipogenic processes, including proliferation, differentiation, lipid droplet formation, and specific genes’ activation in UFA synthesis [71,72,73]. lncRNAs possess the ability to modulate the transcriptional activity of downstream molecules by interacting with proteins and directing protein complexes to specific DNA sequences [37,43]. This transcriptional regulation can control gene expression through both cis-acting and trans-acting mechanisms [37,43]. Studies have demonstrated that the lncRNA ADANR exhibits specific binding affinity towards PA1, facilitating the recruitment of the ML3/4 histone methyltransferase complex and the subsequent activation of C/EBPα in cells, thereby promoting adipogenesis [26]. Heterogeneous nuclear ribonucleoprotein U (hnRNPU) is an essential factor for brown adipocyte development, while the brown adipose tissue-specific lncRNA 1 (lnc-BATE1) acts in trans by interacting with hnRNPU to regulate brown adipogenesis [74]. Furthermore, a recent study revealed that a novel lncRNA, BADLNCR1, can bind to glutaredoxin 5 (GLRX5) through cis-acting. This prevents the binding and activation of CEBPα to the GLRX5 promoter, ultimately resulting in the negative modulation of bovine adipocyte differentiation [75]. This study combined proteomic data with published transcriptomic data to construct a DEL-DEP regulatory network. The results indicate potential interactions between 11 DELs and 12 DEPs in the DEL-DEP cis-regulatory network, including PPP1R2, APOD, and TCONS_00002149; TCONS_00035606 and STK3; TCONS_00171435 and RBM3; TCONS_00100247 and METTL9; TCONS_00081383 and EFL1; TCONS_00023208 and GOLGA2; TCONS_00006766 and GOLIM4; TCONS_00056973 and PLD3; TCONS_00145888 and PPIC; TCONS_00051516 and ALDH2; and TCONS_00075216 and FZD7. The DEL-DEP trans-regulatory network shows potential interactions between two DELs and nine DEPs. Among these interactions, TCONS_00069661 interacts with ADAMTSL1, ECE1, RCN3, FABP4, GFPT2, TGM2, GPAT3, and ANXA8L1, while TCONS_00050038 interacts with MSRA. We hypothesize that these DEPs and DELs in the DEL-DEP regulatory network may be the key proteins and lncRNAs that regulate UFA synthesis, as screened in this study (Figure 9). It is noteworthy that lncRNAs can exert diverse regulatory effects on the same gene at both the transcriptional and protein levels [72,75,76]. For instance, a targeting relationship has been identified between TCONS_00069661 and FABP4 in both the DEL-DEM co-expression network from our previous study [30] and the DEL-DEP trans-regulatory interaction network established in this study.

## 5. Conclusions

In this study, the protein expression profiles of bovine adipocytes were investigated using RNA interference and non-interference with *ACSL1*. The differential expression analysis identified 1428 DEPs, and the subsequent enrichment analysis screened 50 DEPs potentially involved in UFA synthesis. The interaction network of these 50 DEPs revealed the potential regulatory patterns of the proteins in UAF-related pathways. In addition, the DEL-DEP regulatory network was constructed through combined transcriptomic and proteomic analysis, which indicated that 21 DEPs and 13 DELs could be pivotal genes involved in the biosynthesis of UFAs. This valuable resource offers a genetic foundation by which to enhance the nutritional quality of beef and promote consumer health in the future.

## Figures and Tables

**Table 1:** Targets sequence of siRNA for bovine *ACSL1*.

ACSL1 GenBank No.	siRNA No.	Target Sequence
NM_001076085.1	ACSL1 siRNA-1	GGATAGAGGAGTACCTGTA
NM_001076085.1	ACSL1 siRNA-2	CCCTATGAATGGCTTTCAT
NM_001076085.1	ACSL1 siRNA-3	ACTCTTCTCTATCGACAAT

**Table 2:** Liquid chromatography elution gradient table.

Time (min)	Flow Rate (nL/min)	Mobile Phase A (%)	Mobile Phase B (%)
0	600	94	6
2	600	90	10
45	600	70	30
48	600	65	35
50	600	50	50
51	600	0	100
60.5	600	95	5
61.5	600	95	5
62	600	5	95
67	600	5	95
70	600	95	5

**Table 3:** Protein sample test results.

Sample	Protein Concentration	Total Protein
NC-ACSL1-1	1.10	132.00
NC-ACSL1-2	0.99	118.80
NC-ACSL1-3	1.05	126.00
si-ACSL1-1	0.46	55.20
si-ACSL1-2	0.42	50.40
si-ACSL1-3	0.39	42.90

**Table 4:** Overview of protein identification.

Classification	Number
Total spectra	400,282
Matched spectrum	140,710
Peptide	35,802
Identified protein	3558

**Table 5:** DEP-enriched UFA synthesis-related signaling pathways.

Map ID	Map Title	*p*-Value	Annotated DEPs for This Pathway
map04024	cAMP signaling pathway	0.015	E1BC59, A0A3Q1LUJ8, A7MBH9, F1N319, Q3SWW9, Q3T0E7, P46196, A0A3Q1MNN7, A0A3Q1M647, P63097, A0A3Q1M066, A1XG22, D3K0R6, P23727
map04921	Oxytocin signaling pathway	0.019	P60712, Q3SYU2, E1BC59, A7MBH9, F1N319, Q3SWW9, Q3T0E7, P46196, A0A3Q1MNN7, A0A3Q1M647, P63097, E1BA29, A0A3Q1MB84, A0A3Q1M066, A5D7T5
map00071	Fatty acid degradation	0.0228	P48818, Q17QI3, A0A3S5ZP98, A0A3Q1NDR1, Q3T0R7, F1N1M7, Q3ZC42, A0A3Q1LZB8, P20000, Q2KJH9
map00561	Glycerol metabolism	0.0289	A0A3S5ZP98, A0A3Q1LZB8, P20000, Q2KJH9, A0A3Q1MGZ4, E1BGF8
map04910	Insulin signaling pathway	0.0416	A0A3Q1M168, A0A3Q1M0F0, F1MDS3, Q3SWW9, P00515, Q3T0E7, P46196, A0A3Q1MNN7, E1BME6, F1MU24, P00514, B0JYK4, A2VDU0, I7CLV3, F1MQ96, E1BBJ7, P23727
map04923	Regulation of lipolysis in adipocytes	0.0459	A7MBH9, P63097, I7CLV3, F1MHQ4, P23727

## Data Availability

The original contributions presented in this study are included in the article/Supplementary Materials. The mass spectrometry proteomics data have been deposited at the ProteomeXchange Consortium (https://proteomecentral.proteomexchange.org/ (accessed on 19 March 2024)) via the iProX partner repository with the dataset identifier PXD050702. Further inquiries can be directed to the corresponding author.

## Supplementary Materials

The following supporting information can be downloaded at: https://www.mdpi.com/article/10.3390/antiox13060641/s1, Figure S1: Length distribution of peptide sequences; Figure S2: Number distribution of unique peptides; Figure S3: Protein molecular weight distribution; Table S1: Information on DEPs; Table S2: Functional GO terms enriched by upregulated DEPs; Table S3: Functional GO terms enriched by downregulated DEPs; Table S4: Upregulated DEPs’ enriched signaling pathways; Table S5: Downregulated DEPs’ enriched signaling pathways; Table S6: Data for the DEL-DEP (lnRNA cis-target) regulatory network; Table S7: Data for the DEL-DEP (lnRNA trans-target) regulatory network.
