# Supplementary material for: Proteome Analysis Related to Unsaturated Fatty Acid Synthesis by Interfering with Bovine Adipocyte ACSL1 Gene
Source: Antioxidants (Basel). 2024 May 24;13(6):641. doi: 10.3390/antiox13060641 (PMC11200461; doi:10.3390/antiox13060641)
Supplement: Supplementary file 1 [file antioxidants-13-00641-s001.zip › Supplementary files/Supplementary Figure.pdf]

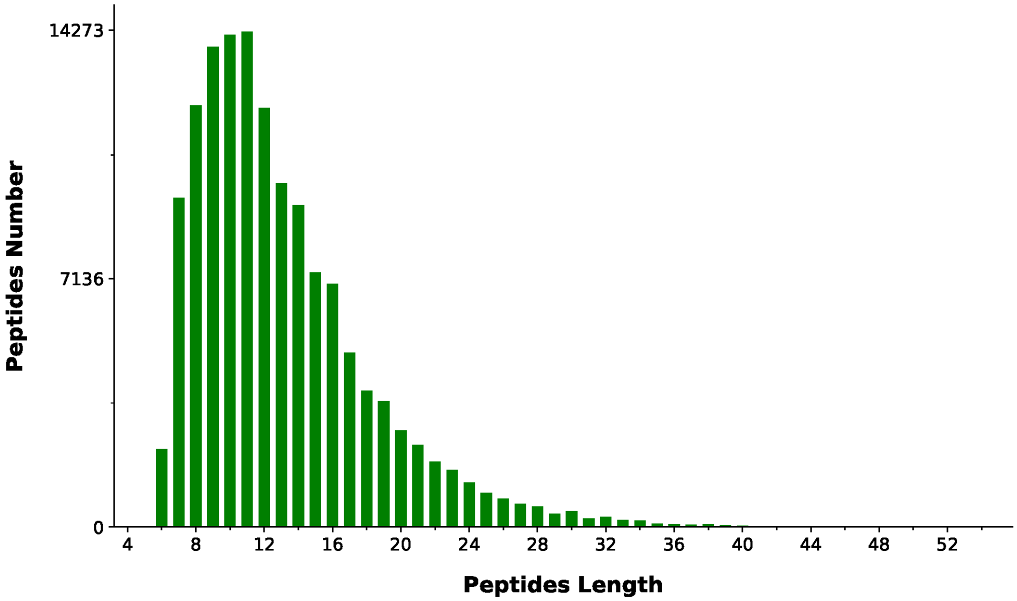

Figure S1. Length distribution of peptide sequences. The abscissa is the number of amino acid residues in the peptide, and the ordinate is the number of peptides of this length.

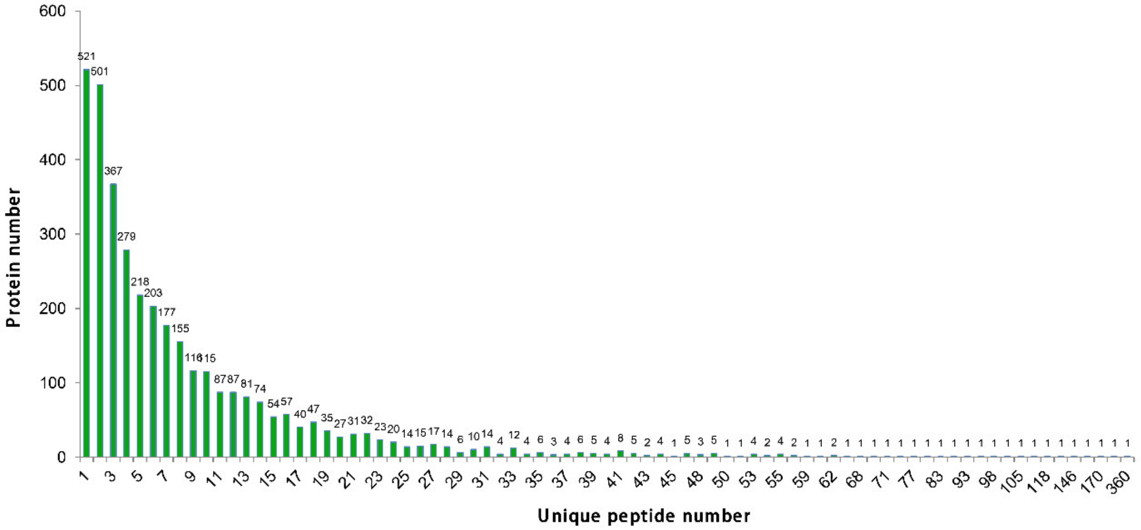

Figure S2. Number distribution of unique peptides. The abscissa is the number of unique peptides, and the ordinate is the number of proteins containing the unique peptide.

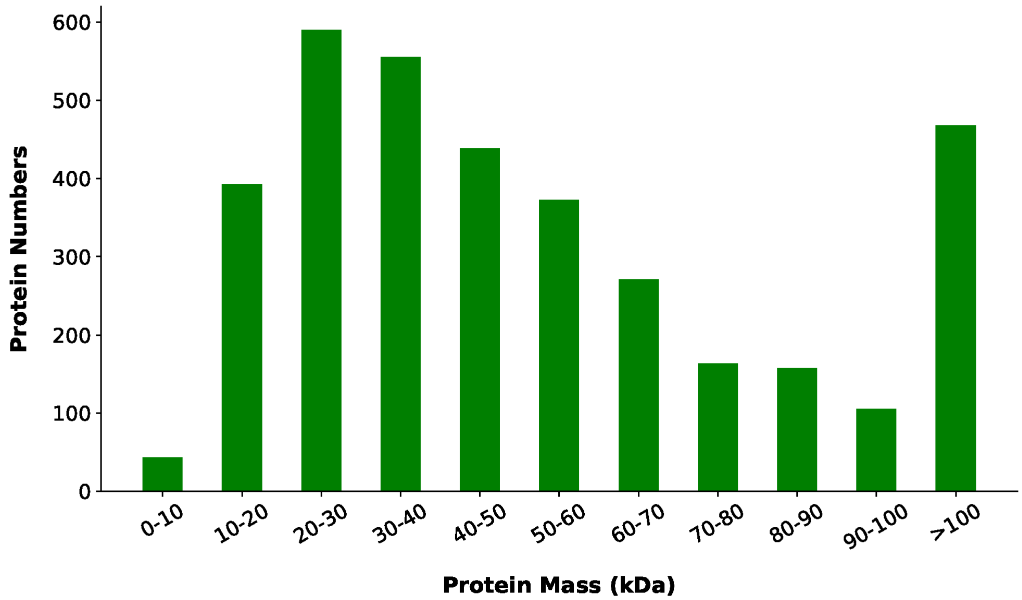

Figure S3. Protein molecular weight distribution. The abscissa is the molecular mass of the identified protein (unit: kilodalton, kDa), and the ordinate is the number of the identified protein.
